# Time course of recovery of different motor functions following a reproducible cortical infarction in non-human primates

**DOI:** 10.3389/fneur.2023.1094774

**Published:** 2023-02-09

**Authors:** Akito Kosugi, Yosuke Saga, Moeko Kudo, Masashi Koizumi, Tatsuya Umeda, Kazuhiko Seki

**Affiliations:** ^1^Department of Neurophysiology, National Institute of Neuroscience, National Center of Neurology and Psychiatry, Tokyo, Japan; ^2^Department of Integrated Neuroanatomy and Neuroimaging, Graduate School of Medicine, Kyoto University, Kyoto, Japan

**Keywords:** non-human primate (NHP), stroke, common marmoset, photothrombosis, visually-guided reaching

## Abstract

A major challenge in human stroke research is interpatient variability in the extent of sensorimotor deficits and determining the time course of recovery following stroke. Although the relationship between the extent of the lesion and the degree of sensorimotor deficits is well established, the factors determining the speed of recovery remain uncertain. To test these experimentally, we created a cortical lesion over the motor cortex using a reproducible approach in four common marmosets, and characterized the time course of recovery by systematically applying several behavioral tests before and up to 8 weeks after creation of the lesion. Evaluation of in-cage behavior and reach-to-grasp movement revealed consistent motor impairments across the animals. In particular, performance in reaching and grasping movements continued to deteriorate until 4 weeks after creation of the lesion. We also found consistent time courses of recovery across animals for in-cage and grasping movements. For example, in all animals, the score for in-cage behaviors showed full recovery at 3 weeks after creation of the lesion, and the performance of grasping movement partially recovered from 4 to 8 weeks. In addition, we observed longer time courses of recovery for reaching movement, which may rely more on cortically initiated control in this species. These results suggest that different recovery speeds for each movement could be influenced by what extent the cortical control is required to properly execute each movement.

## Introduction

The most common deficit after stroke is motor impairment (1), and ~60% of stroke patients do not completely recover their upper limb function, such as target-reaching and hand-grasping (2). The relationship between the extent of the lesion and the degree of deficits is well established (3, 4); however, factors that determine the speed of recovery remain uncertain. A lack of an optimal animal model of stroke for reproducing upper limb motor deficits in terms of both the extent and recovery process is a major limitation that has hindered the development of an effective therapeutic intervention.

Although several stroke models using rodents have been established (5), non-human primate (NHP) models remain indispensable (6–9) because NHPs provide an advantage over rodents when reproducing the reaching and grasping movements of a human stroke patient. First, the musculature and functionality of the hand differ between rodents and primates [for review see (10)]. For example, the intrinsic hand muscles have vast differences in anatomy between the two animals. Thus, finger individualization is less frequently measured in rodents than in primates (11, 12). Second, the cortical visual pathway for visually guided behaviors is developed in primates (13). For example, neurons in the parietofrontal cortex are activated during visually guided reaching (14–16), and lesions in this pathway cause deficits in reaching performance (17–19). In contrast, rodents primarily use olfaction to identify the location of a target, and thus, reaching toward a target is guided more by olfaction than by vision (20–22). In addition, recent studies demonstrated the advantage of NHPs over rodents in terms of cellular divergency in the central nervous system (CNS), with an important implication in the context of inflammation (23, 24).

Therefore, the use of existing NHP models (8, 25) is advantageous to using the rodent model for stroke research. However, NHP models show significant inter-individual variability in the extent and recovery time course of outcome measures, which is largely due to the technical complexity of applying an infarction and the limited availability of animals to refine such techniques. For example, in an anterior choroidal artery occlusion model, only 60% of animals showed neurological impairment (26). In an internal capsular infarct model, the duration of recovery varied among animals (27, 28). Such inter-animal variability can be compensated for by increasing the number of animals in the case of rodent models, whereas this is more challenging for NHP models. Consequently, NHP stroke models have been less popular for use in stroke research to date (29, 30).

In this study, we aimed to overcome this problem by using a photothrombotic approach (31–38), which involves the intravenous administration of photosensitive dye, followed by irradiation of the cerebral cortex with green light (31). The irradiation triggers the formation of a blood clot that occludes the vessels (5, 39, 40). Because the area of infarction can be controlled by irradiation light, this method has the advantages of high reproducibility and low mortality (7, 8, 39–41).

The purpose of this study was to create a cortical infarction using photothrombosis over the motor cortex of NHPs to establish a reproducible deficit in the reaching and grasping task. We then characterized the time courses of recovery of the reaching and grasping functions.

## Materials and methods

### Animals

Four adult common marmosets (*Callithrix jacchus*, aged 3–6 years, three males and one female, weighing 300–550 g) were used in the present study (Table 1). All interventions and animal care procedures were performed in accordance with the institutional guideline for animal experiments and the National Institutes of Health Guide for the Care and Use of Laboratory Animals. All experiments were approved by the experimental animal committee of the National Institute of Neuroscience.

**Table 1:** Marmosets used in the study.

**Subject**	**Sex**	**Age (years)**	**Lesion volume [mm^3^]**
Monkey K	male	3.4	43.5
Monkey M	female	5.2	38.5
Monkey P	male	6.0	41.1
Monkey U	male	5.0	45.4

### Surgical procedure

We created an infarction over the unilateral motor cortex using Rose Bengal, which is a light-sensitive dye, according to a previous study (36). A 3 mm diameter liquid light guide connected to the light source (Spectra X light engine, Lumencor, Beaverton, OR, USA) was placed 16 mm above the motor cortex, which was identified with the aid of a stereotaxic atlas (42). After intravenous injection of Rose Bengal (20 mg/kg), green light (542.5–557.5 nm) was irradiated for 5 min at a light intensity of 48 mW. All surgeries were performed under anesthesia induced by intramuscular induction of ketamine hydrochloride (20 mg/kg) and maintained by inhalation of isoflurane (2%−3%). Atropine sulfate, antibiotics, analgesics, and dexamethasone were used to prevent postsurgical infection, pain, and edema. Mannitol (1.0–1.5 mL/h) was infused if necessary to reduce intracranial pressure during surgery. Antibiotics and analgesics were injected twice (morning and afternoon) daily for 5 days following creation of the lesion.

To expose the green light, we performed a craniotomy. The skull was opened between interaural, 5–15 mm anteroposterior (AP) and 2–12 mm mediolateral (ML) from the midline. A probe for light exposure was then placed on the opened skull at the center, 8 mm AP and 4 mm ML. To limit irradiation of the light, a perforated aluminum cover (3 mm AP × 8 mm ML) was placed onto the brain. We have already confirmed that craniotomy itself does not alter the spontaneous in-cage behavior after surgery (26).

### Behavioral assessments

#### Marmoset neurologic score (MNS)

To evaluate the natural recovery process of sensorimotor functions, neurological status was evaluated using a neurological score described previously (26). First, we recorded the spontaneous natural behavior of the marmosets in the home cage *via* a video camera placed in front of their cages. Two experienced experimenters then carefully inspected the recorded video and judged the absence (score = 1) or presence (score = 0) of 18 abnormal behavioral signs in their home cage (Table 2). We omitted several test items from the original test (26) that required retrieval of the marmoset from the home cage (e.g., the “stick” and “limb stimuli” tests). The maximum score was 18, and a lower score indicated greater motor impairment. The tests were performed before and 1, 2, 7, 14, 21, and 28 days after creation of the lesion.

**Table 2:** Modified marmoset neurologic score.

**General evaluation**	**Hemilateral evaluation**
Stays in back of the cage	Body tilting
Stays still for 1 min	Head tilting
Cannot stand in the perch	Hand waving
Circling behavior	Repeated touching before grasp cage bars
Palpebral ptosis	Hand crossing the chest
No jumping from cage wall	Hand slipping from the cage bars
No rearing without hand support	Hand dangling from the cage bars
	Hand neglect during feeding
	Foot slipping
	Foot dangling
	Dropping crumbs

Total score: 18 points (general: 7 points; hemilateral: 11 points).

#### Pellet-reaching task

**Figure 1:**
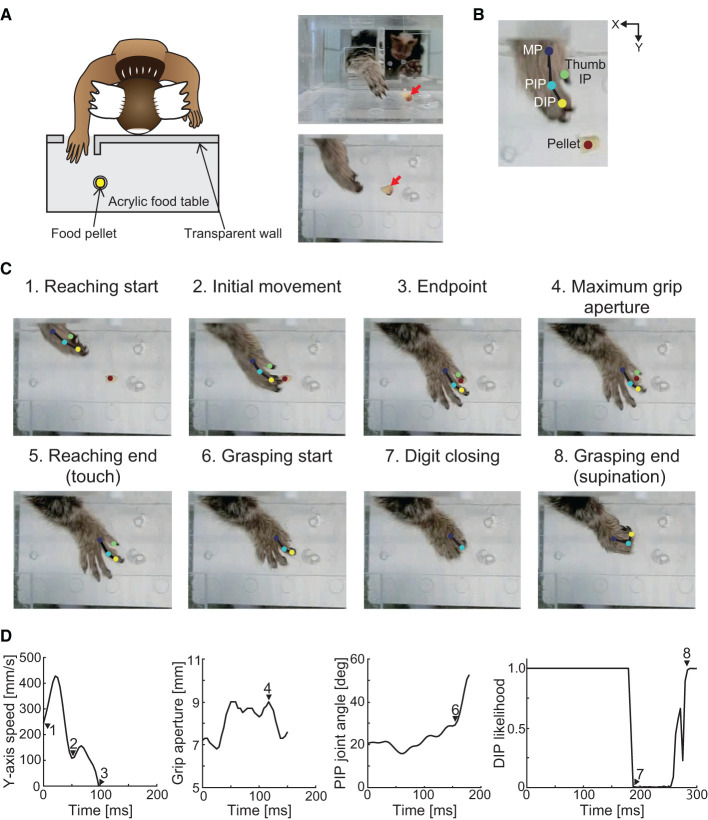
Schematic of the experimental setup and definition of the movement phase. **(A)** Experimental setup. Red arrows in the right images indicate the position of the food pellet. **(B)** Positions of the tracking using DeepLabCut. Two-dimensional positions of the distal interphalangeal (DIP), proximal interphalangeal (PIP), and metacarpophalangeal (MP) joints of the index finger, interphalangeal (IP) joint of the thumb, and food pellet were tracked. **(C)** Definition of the movement phases. The reaching movement phase was defined as the time at which the MP joint of the index finger passed the opening (“reaching start”) to the time when the hand touched the food pellet (“reaching end”). The grasping movement phase was defined as the time at which the index finger started to flex (“grasping start”) to the time when the wrist began to supinate (“grasping end”). **(D)** Y-axis movement speed, grip aperture, PIP joint angle, and likelihood estimates of the index finger DIP joint position for each data point using DeepLabCut. Each number corresponds to those in **(C)**.

To evaluate the influence of cerebral ischemia on forelimb sensorimotor function, we trained three marmosets (Monkeys K, M, and P) to perform a pellet-reaching task. A clear acrylic food table was attached to the cage, and the monkeys were forced to use their impaired limb (Figure 1A). The table was 180 mm in length and 60 mm in width and was attached 80 mm above the floor of the cage. A transparent wall (180 mm in length and 65 mm in depth) was attached to the front edge of the table in front of the marmoset. A small opening (a 25 mm square) was located 10 mm from the bottom of the transparent wall, which forced the monkeys to use their affected hand. A sweet treat (4–8 mm in diameter) was placed in a small well on the table (8 mm in diameter, 1 mm in depth, and 20 mm from the opening). Two marmosets performed the task with the right hand (Monkeys M and P) and one marmoset performed the task with the left hand (Monkey K). We defined the success rate as a percentage of the ratio between the number of successful retrievals with the affected hand and the number of reaches with the affected hand. All marmosets were trained for 20 min for 5 days per week for up to 7 weeks until their baseline performance plateaued (>80% success rate). The tests were performed before and 1, 2, 4, and 8 weeks after creation of the lesion. In a single test, the marmosets attempted 20–40 food pellet retrievals using the affected hand over 10 min. If the animals did not use the affected hand within 5 min, the success rate was recorded as zero.

To evaluate hand kinematics, recordings using a high-speed camera were acquired during the task before and 4 and 8 weeks after creation of the lesion. Two-dimensional positions of the affected hand were recorded using a high-speed camera (EX−100F; CASIO COMPUTER, Tokyo, Japan). The camera was operated at 240 frames/s at a 640 × 480-pixel resolution. The camera was placed 230 mm from the table horizontally to ensure that the animal's hand was tracked throughout reaching and retrieving movements.

Most tests were performed in the home cage. However, some sessions were performed in the breeding room. In such sessions, we used a cage of identical size as that of the home cage.

#### Processing of video recordings

To quantify the movement trajectories during pellet-reaching, we measured the two-dimensional positions of the hand and pellets using DeepLabCut (version 2.2b8) (43, 44). First, we annotated the two-dimensional positions of the distal interphalangeal (DIP), proximal interphalangeal (PIP), and metacarpophalangeal (MP) joints of the index finger, interphalangeal (IP) joint of the thumb, and the food pellet (Figure 1B). Next, we trained two deep neural networks (i.e., a right-hand network and a left-hand network) based on transfer learning of the pre-trained network (ResNet50). In the right-hand network, we labeled a total of 1,500 images that were randomly selected from 17 trials (two different targets at three different time points, one or two trials each) in two animals (Monkeys M and P). In the left-hand network, we labeled a total of 1,199 images that were randomly selected from 12 trials (two different targets at three different time points, two trials each) in one animal (Monkey K). The ratios of the training data to the annotated data were 0.95, and the training iterations were 1,030,000.

Once the networks were trained, we performed separate validation procedures for the two networks. The train and test errors were as follows: the right-hand network was 2.51 pixels and 2.12 pixels, respectively, and the left-hand network was 2.09 pixels and 1.95 pixels, respectively. The model provided likelihood estimates for each tracking result at each time point. We regarded the tracking result with a likelihood of < 0.7 as an occlusion. We removed results with a likelihood between 0.7 and 0.95 and performed linear interpolation. The ratio of the removed frames to total frames was < 1%.

We then converted the tracking results into actual two-dimensional coordinates using the four landmarks on the table for which the two-dimensional coordinates were determined previously. The converted data were low-pass filtered at 30 Hz in each coordinate axis, and the hand position, movement speed, grip aperture, and finger-joint angle were calculated using the filtered data. Specifically, the hand position was calculated from the two-dimensional coordinates of the index finger MP joint, and movement speed was calculated by the differential of the Y-coordinate positions of the index finger MP joint. Grip aperture was calculated according to the Euclidean distance between the two-dimensional coordinates of the IP joint of the thumb and the DIP joint of the index finger. Using the two-dimensional coordinates of the MP, PIP, and DIP joints, we calculated the horizontally projected angle of the index finger PIP joint.

#### Kinematic analysis

Using hand position, movement speed, finger-joint angle, and likelihood estimates for each data point, we defined the movement phases (Figures 1C, D). We defined eight discrete movement task epochs from the processed video recordings. The “reaching start” was defined as the time at which the MP joint passed the opening when the likelihood estimate of the MP joint exceeded 0.95. The “initial movement” phase was defined as the period from the “reaching start” to the first local minimum in movement speed. The “endpoint” was defined as the time at which the Y-axis movement speed dropped below the speed threshold (0 mm/s) for at least 25 ms. The definitions of “initial movement” and “endpoint” were adopted from the visually guided reaching task in human stroke patients (45). The “maximum grip aperture” was defined as the time at which the grip aperture between the thumb and index finger was the largest. The “reaching end” was defined as the time at which the hand touched the food pellet when the distance between the MP joint and the food pellet was the smallest. The “grasping start” was defined as the time at which the index finger started to flex when the angular velocity of the PIP joint angle first exceeded 5% of the peak angular velocity. Because wrist supination occurred as soon as the food pellet was grasped, “grasping end” was defined as the time at which the index finger DIP joint could be seen from above after the digits closed, when the likelihood estimate of the index finger DIP joint exceeded 0.95.

The data were visually inspected, and data were discarded when the marmoset failed to perform a successful reaching movement, or when the animal was unable to touch, displaced, or dropped the pellet. The ratios of reaching failures to grasping failures at each time point are shown in Table 3. Data in which the grasping movement took longer than 1 s were also excluded from the statistical analysis. Eventually, we analyzed five reaching and grasping movements for each time point.

**Table 3:** Details of the pellet-reaching task.

**Subject**	**Time point**	**Success ratio (%)**	**Failure ratio (%)**		
			**Reaching failure**	**Grasping failure**	**Pull-back failure**
Monkey K	Pre	90	0	10	0
	4 weeks	36	23	23	18
	8 weeks	39	13	17	31
Monkey M	Pre	95	0	5	0
	4 weeks	45	37	13	5
	8 weeks	96	0	4	0
Monkey P	Pre	81	0	5	14
	4 weeks	31	44	10	15
	8 weeks	20	15	50	15

To evaluate reaching performance in detail, we used two further movement parameters that represent the initial motor response and feedback corrections of the visually guided reaching task, which are used in human stroke patients (45). An initial movement direction error that represents the initial motor response was defined as the angular deviation between a straight line from the MP joint position at the “reaching start” to the target position and a vector from the MP joint position at the “reaching start” to the “initial movement.” The number of speed maxima that represents the feedback corrections was defined as the number of Y-axis movement speed maxima between the “reaching start” and the “endpoint.” We also evaluated the grasping performance in detail to measure the grasping time and the maximum grip aperture. Grasping time was defined as the total time from “grasping start” to “grasping end.” Maximum grip aperture is a clinically relevant outcome measure of functional impairment in human patients (46), and has been demonstrated to be altered in marmosets after lesion of the cortical visual pathway (13, 19). Following the methods of previous studies, we defined the maximum grip aperture as the maximum value of the grip aperture between the thumb and index finger before “grasping start” (13, 19).

### Histology

#### Immunohistochemistry

After the marmosets had performed all the experiments, including the pre- and post-lesion sessions, they were deeply anesthetized and transcardially perfused with 4% paraformaldehyde in phosphate buffer (pH 7.4). The fixed brains were removed from the skull, postfixed in the same fresh fixative overnight at 4°C, and placed into 0.1 M phosphate buffer (pH 7.4) containing 30% sucrose. The brains were then cut along the coronal plane into 50 μm thickness slices using a freezing microtome. One section out of six was immediately mounted for thionin staining. For immunohistochemistry, adjacent sections were incubated with a mouse monoclonal antibody for glial fibrillary acidic protein (1:1,500 dilution; Sigma-Aldrich, St. Louis, MO, USA) or a rabbit polyclonal antibody for Iba-1 (1:4,000 dilution; WAKO Pure Chemical Industries, Osaka, Japan). Secondary biotinylated anti-mouse (1:200 dilution; Vector Laboratories, Burlingame, CA, USA) or biotinylated anti-rabbit (1:200 dilution; Vector Laboratories) antibodies were also used. Immunoreactive signals were visualized using the ABC Staining Kit (Vector Laboratories) with 3,3'-diaminobenzidine. All stained images were acquired using an inverted microscope (BZ-X700, Keyence, Osaka, Japan).

#### Identification of lesion area

**Figure 2:**
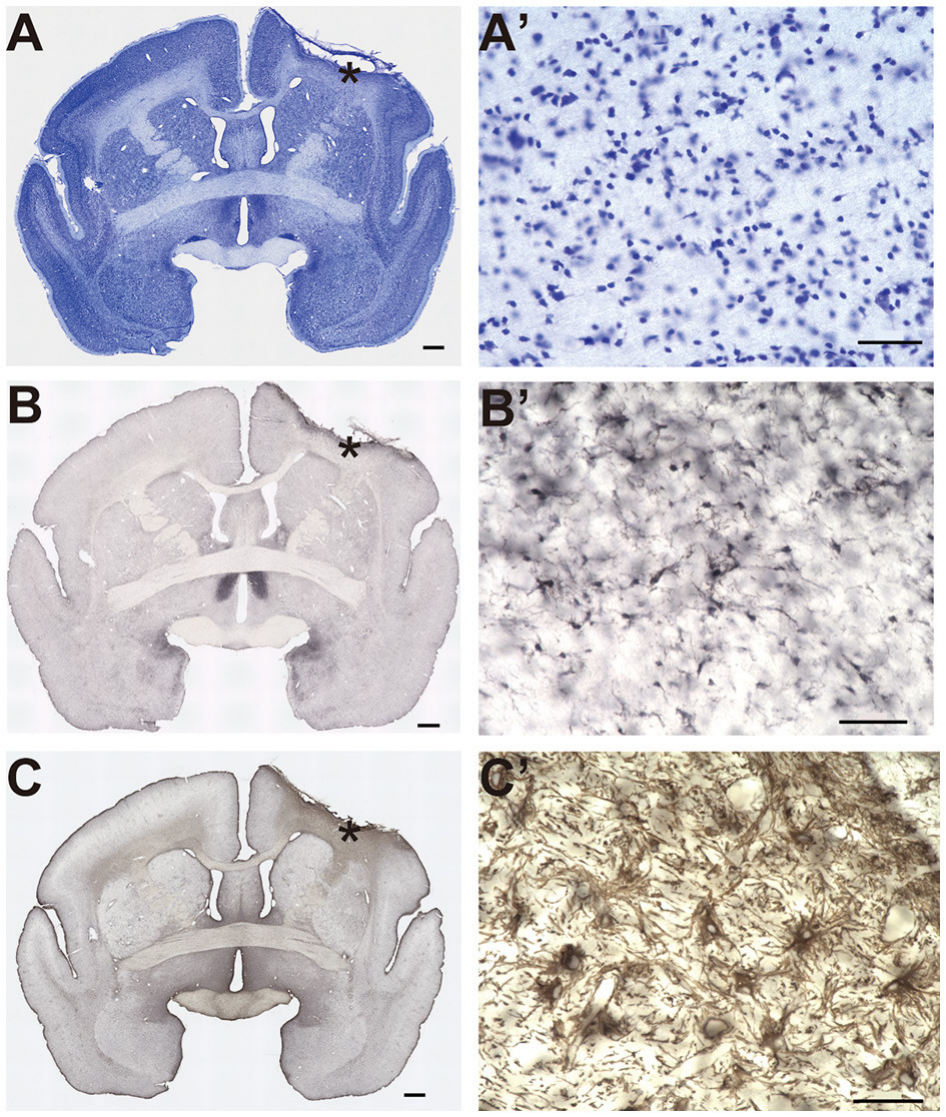
Histological identification of the lesion area. **(A–C)** Representative image of Nissl staining **(A)**, Iba-1 immunostaining **(B)**, and glial fibrillary acidic protein immunostaining **(C)**. Asterisks indicate the locations of the higher magnification view shown in **(A'–C')**. Scale bar, 1 mm for **(A–C)**, and 50 μm for **(A'–C')**.

**Figure 3:**
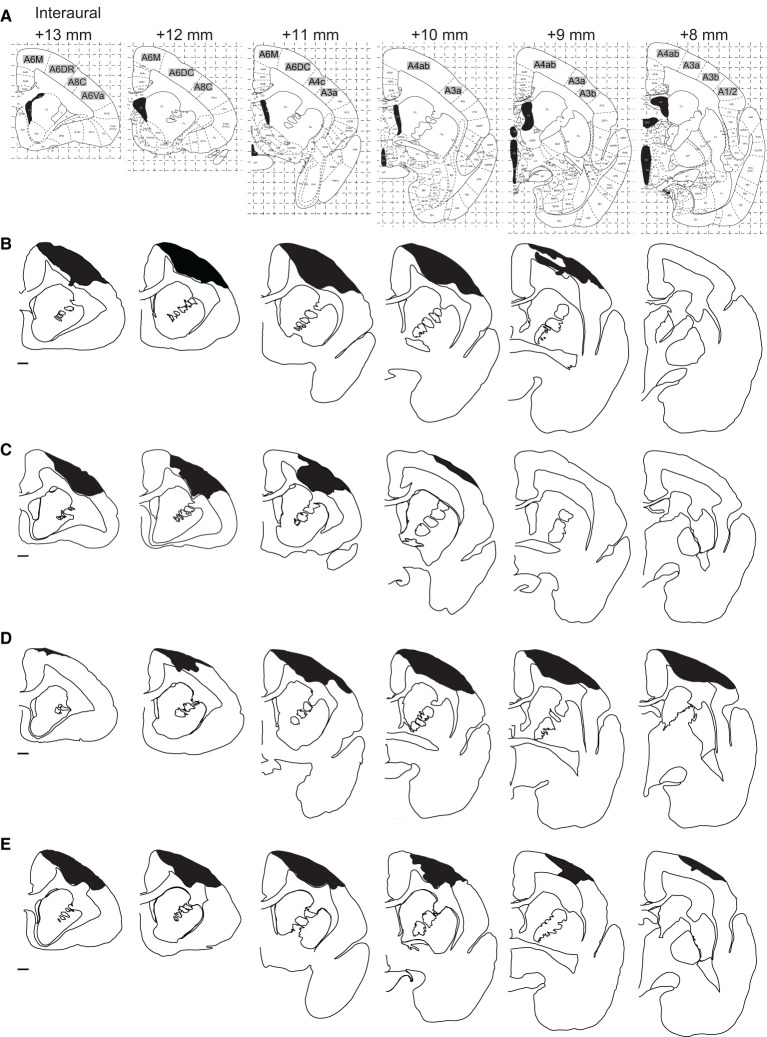
Distribution of the lesion area. **(A)** The corresponding coronal sections of the marmoset brain atlas (42). **(B–E)** Serial coronal sections of the lesion area in Monkey K **(B)**, Monkey M **(C)**, Monkey P **(D)**, and Monkey U **(E)**. Black hatched areas indicate the lesion area identified by Iba-1 immunostaining. Only the hemispheres ipsilateral to the lesion site are shown at intervals of 1 mm. Scale bar, 1 mm.

To identify the lesioned cortical area induced by photothrombosis, we detected the area showing an inflammatory response (Figures 2B, 3). First, we selected sections showing an inflammatory response based on Iba-1 immunochemistry with a 300 μm space between serial sections. We then manually traced the cortical area labeled with the Iba-1 antibody. Because several cortical structures were lost under the irradiation area, we estimated the area of the lost cortical structure by tracing the interhemispheric difference between the contralesional hemisphere and the ipsilesional hemisphere in each section. From these tracings, we calculated the total volume of the lesioned areas using ImageJ (National Institutes of Health, MD, USA) using the following formula:


$$\begin{array}{l} volume=d\sum \left(S_{contra}-S_{ipsi}+S_{Iba-1}\right)\left(1\right), \end{array}$$

where d is the distance between sections (300 μm), and *S*_*contra*_, *S*_*ipsi*_, and *S*_*Iba*−1_ are the traced areas of the contralesional hemisphere, ipsilesional hemisphere, and Iba-1-positive cortical structure, respectively.

### Experimental design and statistical analysis

To assess the differences in the time course of recovery between subjects, a two-way analysis of variance with aligned rank transform [(ART-ANOVA); (47, 48)] was performed for each index, with “time point” (“Pre,” “4 weeks,” and “8 weeks”) and “subject” (Monkeys M, K, and P) as between-subject factors. *Post-hoc* analyses were performed using Holm-Bonferroni correction for multiple comparisons. The level of significance was set at α = 0.05. All data analyses and statistical tests were performed using MATLAB 2018b (MathWorks, Natick, MA, USA).

## Results

### Area and extent of the lesion

An example of the extent of the lesioned cortical area is shown in Figures 2A–C (Monkey P). In this subject, we found dense cell infiltration under the irradiation area (Figure 2A'). Clear microglia accumulation within the same area indicated an inflammatory response (Figure 2B'). Furthermore, an aggregation of reactive astrocytes was observed within the same area (Figure 2C'). Taken together, these results suggested that the extent of the lesion encompassed the infarction under the irradiation area.

We then compared the area and extent of the lesion among the four animals. The area immunostained by Iba-1 is shown in black hatched area in Figure 3. We found that Brodmann's areas 6M, 6DR, 6DC, 4c, and 4ab were the locus of damage with the highest probability, according to the marmoset brain atlas [(42); Figure 3A]. Area 4 corresponds to the primary motor cortex (M1), area 4b corresponds to the forelimb movement representation in the M1 (42, 49–51), and areas 6M, 6DR, and 6DC correspond to the supplementary motor area and rostral and caudal area of the dorsal premotor cortex (PMd), respectively (42, 52). Therefore, we concluded that the lesioned area was mainly localized to the motor-related cortical areas. In addition, two animals (Monkeys P and U) showed ischemic damage in the primary somatosensory cortex [areas 3a and 3b in Monkeys P and U (Figures 3D, E) and area 1/2 in Monkey P (Figure 3D)]. The average lesion volume among animals was 42.1 ± 3.0 mm^3^ (Table 1).

### Time course of behavioral recovery

**Figure 4:**
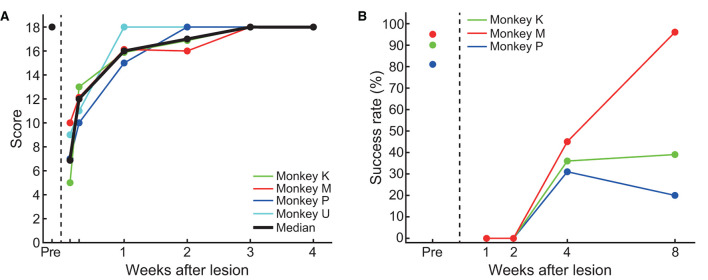
Time course of behavioral recovery. **(A)** Transitional changes in the marmoset neurological score. The black line represents the median value at each time point. **(B)** Transitional changes in the success rate of the pellet-reaching task.

Figure 4A shows the time course of the changes in the MNS score. Before the lesion was created, all animals scored the maximum score (18 points). One day after creation of the lesion, all marmosets showed an expected decrease in the MNS score (median = 7). The score recovered rapidly over the subsequent weeks in all animals. Specifically, from 2 days to 1 week after creation of the lesion, the median score increased from 12 to 16 points. At 3 weeks after creation of the lesion, animals fully recovered and scored the maximum score (18 points).

This systematic recovery time course in all monkeys as measured by the MNS score (Figure 4A) was also supported by the descriptive observations of the daily behaviors of the marmosets in their cages. One day after creation of the lesion, the animals often stayed at the back of the cage, their body and head were tilted, and they frequently let their hand and feet slip or dangle from the cage bars. They also held the cage bars close to their chest using their intact hand. At 1 to 2 weeks after creation of the lesion, several abnormal behaviors continued to be exhibited, such as dangling their hands from the cage bars. However, these abnormal behaviors were not observed from 3 weeks after creation of the lesion.

Figure 4B shows the weekly change in the success rate of the pellet-reaching task. Before the lesion, success rates were >80% in all animals. After creation of the lesion, their performance was completely impaired for 2 weeks (i.e., 0% success rate), which suggested that the lesion drastically affected the function of the contralesional limb. At 2 to 4 weeks after creation of the lesion, the animals started to use their impaired limb to reach and retrieve pellets. However, in contrast to the MNS score (Figure 4A), success rates remained lower (31–45%) than those before creation of the lesion, and recovery varied among animals. For example, one marmoset (Monkey M) showed an improvement in success rate, whereas the other two marmosets (Monkeys K and P) showed little improvement. This heterogeneous recovery among animals is further described in Table 3. At 4 weeks after creation of the lesion, the predominant reason for failed trials was “reaching failure,” where 23–44% (Table 3) of failures were caused by this error. Reaching failures are primarily due to reaching for the pellet in an inappropriate direction, which resulted in their hand not reaching the pellet, based on our visual observations. Another reason for failed trials at this time point was “grasping failure” (10–23% of all failures; Table 3), in which animals exhibited clumsy digit movements, which resulted in the pellet being displaced or dropped.

At 8 weeks after creation of the lesion, we found mixed results in the three monkeys. In Monkeys K and P, the major sources of failures were both “grasping failures” and “pull-back failures” (Table 3 for Monkeys K and P). In pull-back failures, animals were able to touch the pellet but could not bring the pellet to their mouth. In contrast, we found almost complete recovery in Monkey M. Our analysis of the success ratio for the time course of recovery suggests that controlled cortical lesions can produce a reproducible time course of recovery of motor deficits for at least 4 weeks after creation of the lesion.

A comparison between Figures 4A, B illustrates the unique recovery profiles of pellet-reaching and demonstrates a clear contrast to the earlier recovery of the MNS scores. Although the MNS scores recovered completely and reached a plateau at 3–4 weeks after creation of the lesion (Figure 4A) in a highly similar fashion across all animals, the success rate of pellet-reaching did not recover to the pre-lesion rate at 4 (*n* = 3) or 8 weeks (*n* = 2) after creation of the lesion. These results may reflect the superior resolution of the reaching and grasping test for evaluating the recovery of function represented by the lesioned cortical area.

### Reaching kinematics

**Figure 5:**
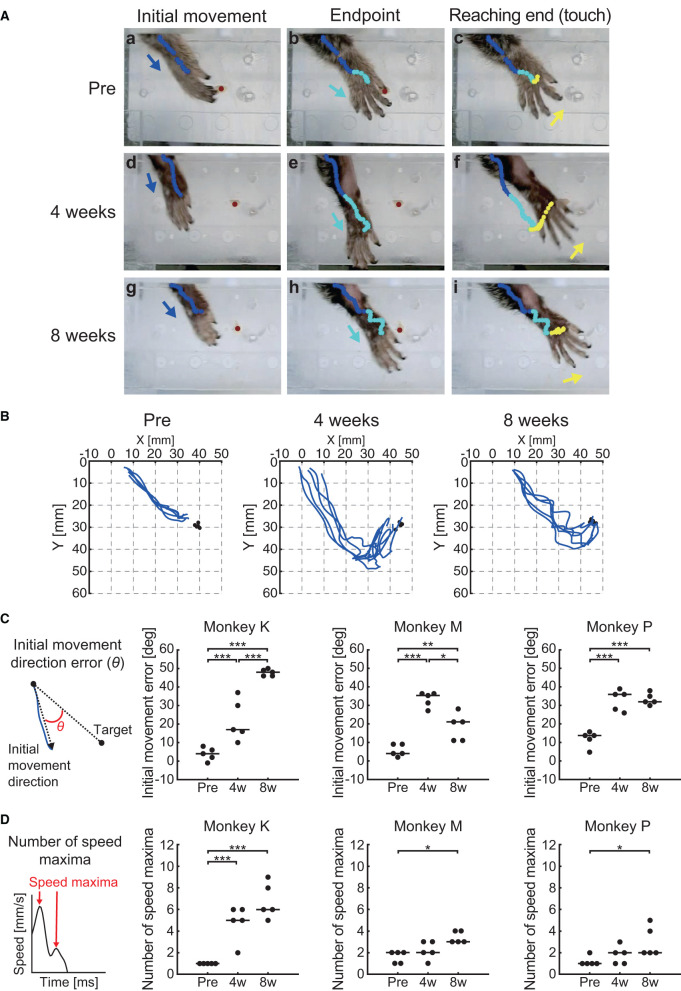
Behavioral results of reaching function. **(A)** Representative images of the reaching movement before, 4 weeks, and 8 weeks after creation of the lesion from one representative marmoset (Monkey M). Blue, cyan, and yellow lines indicate the trajectory of the index finger MP joint between movement phases (blue: from “Reaching start” to “Initial movement”; cyan: from “Initial movement” to “Endpoint”; yellow: from “Endpoint” to “Reaching end”). Blue, cyan, and yellow allows indicate movement direction between movement phases. Red circle indicates the position of the food pellet. **(B)** Typical example of reaching trajectories in the two-dimensional coordinates from one representative marmoset (Monkey M). Black circles indicate the positions at which food pellets were placed. **(C)** Time course of the transition in the initial movement direction error in each marmoset. Left inset indicates schema of the definition in the initial movement direction error, represented by θ. Black lines indicate median value of each time point. *p < 0.05; **p < 0.01; ***p < 0.001. **(D)** Time course of the transition in the number of speed maxima in each marmoset. Left inset indicates schema of the definition in the number of speed maxima. Black lines indicate median value of each time point. *p < 0.05; ***p < 0.001.

Figure 5A shows the hand trajectories during the reaching movement of one representative marmoset (Monkey M). Before creation of the lesion (“Pre”), the animal showed a relatively straight trajectory of the hand toward the target from the beginning to the initial movement phase (Figure 5Aa). This straight trajectory was sustained until the endpoint was reached (Figure 5Ab). The animal then showed small corrective movements before touching the pellet (Figure 5Ac). In contrast, at 4 weeks after creation of the lesion (“4 weeks”), the reaching movement occurred in the incorrect direction at the beginning (Figure 5Ad), which was not corrected even by the end of the reaching movement (Figure 5Ae). The direction was eventually corrected before the hand touched the pellet (Figure 5Af). At 8 weeks after creation of the lesion (“8 weeks”), the misdirection in the initial movement recovered partially but a deviation from the pellet persisted (Figure 5Ag). The hand trajectory showed meandering trajectories (Figure 5Ah, i), which suggested that the animal was correcting the direction of movement during the reaching period. These observations (the trajectory superimposed over the pictures in Figure 5A) are quantitatively represented in the X-Y coordinate and summarized in Figure 5B, illustrating that the hand trajectory significantly drifted along the Y-axis in the overshoot direction at “4 weeks” in contrast to the relatively straight path at “Pre.”

We then quantified these observations by measuring the initial phase of the reaching movement (initial direction error) and the corrective feedback control (number of speed maxima), similarly to the reports measuring sensorimotor impairments in human stroke patients (45, 53). We found significant changes at various measurement points in both the initial direction error (two-way ART-ANOVA, “time point” × “subject” interaction: *F*_(4, 36)_ = 19.6, *p* < 0.0001) and the number of speed maxima (two-way ART-ANOVA, “time point” × “subject” interaction: *F*_(4, 36)_ = 9.0, *p* < 0.0001), as shown in Figures 5C, D. Compared with “Pre”, all animals showed significantly larger initial movement direction errors at “4 weeks” (Figure 5C; Monkey K: *p* = 0.0002, Monkey M: *p* < 0.0001, and Monkey P: *p* = 0.0001) and “8 weeks” (Figure 5C; Monkey K: *p* < 0.0001, Monkey M: *p* = 0.0082, Monkey P: *p* = 0.0001). Similarly, the results of the number of speed maxima showed significantly more corrective movements at “8 weeks” than at “Pre” (Figure 5D; Monkey K: *p* < 0.0001, Monkey M: *p* < 0.05, Monkey P: *p* < 0.05). Only one marmoset (Monkey K) made significantly more corrective movements at “4 weeks” than at “Pre” (Figure 5D; *p* < 0.0001). These results indicated that the impairment in reaching trajectory is characterized by an increase in initial movement direction errors and greater corrective feedback control, which were both comparable to observations in human stroke patients (45, 53). Neither parameter recovered to presurgical levels, even 8 weeks after creation of the lesion.

### Grasping function

**Figure 6:**
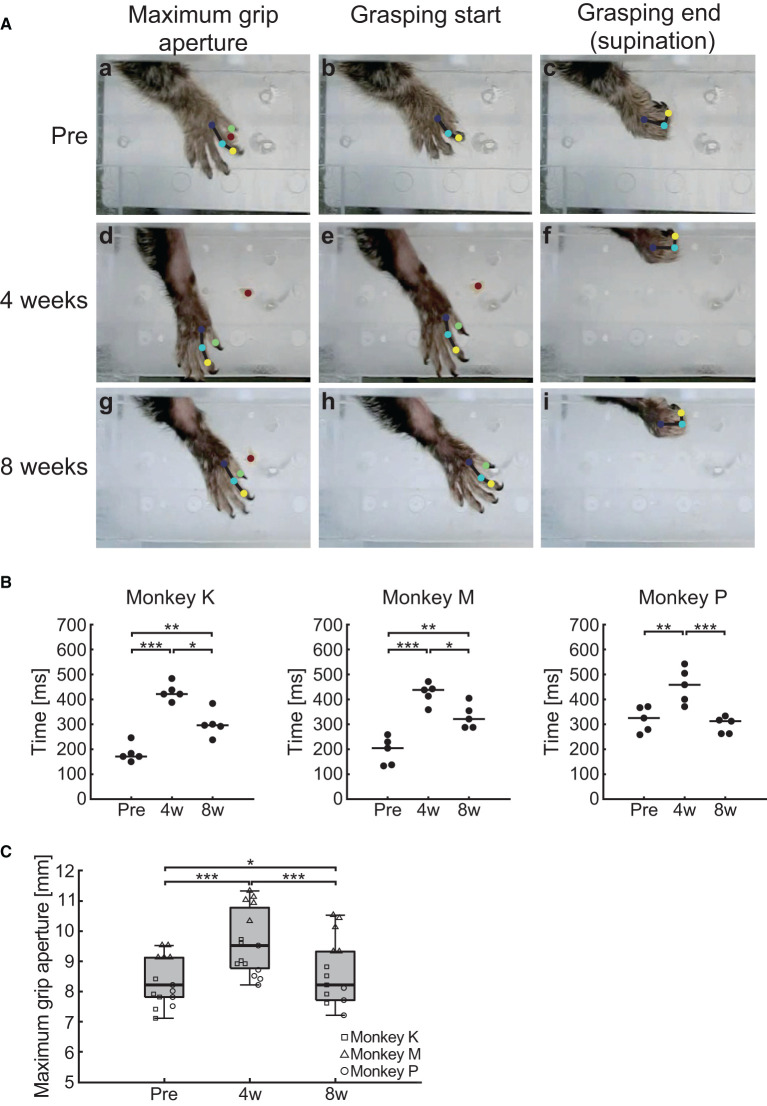
Behavioral results of grasping function. **(A)** Representative images of the grasping movement before, 4 weeks, and 8 weeks after creation of the lesion from one representative marmoset (Monkey M). Blue, cyan, yellow, and green circles indicate the position of the index finger MP, PIP, and DIP joint, and the thumb IP joint, respectively. Red circle indicates the position of the food pellet. **(B)** Time course of the transition in the grasping time in each marmoset. Black lines indicate median value of each time point. *p < 0.05; **p < 0.01; ***p < 0.001. **(C)** Time course of the transition in the maximum grip aperture. Box plots indicate the median (black line in the box), interquartile range (IQR; gray box), and the lowest and highest data (error bars). *p < 0.05, ***p < 0.001.

**Figure 7:**
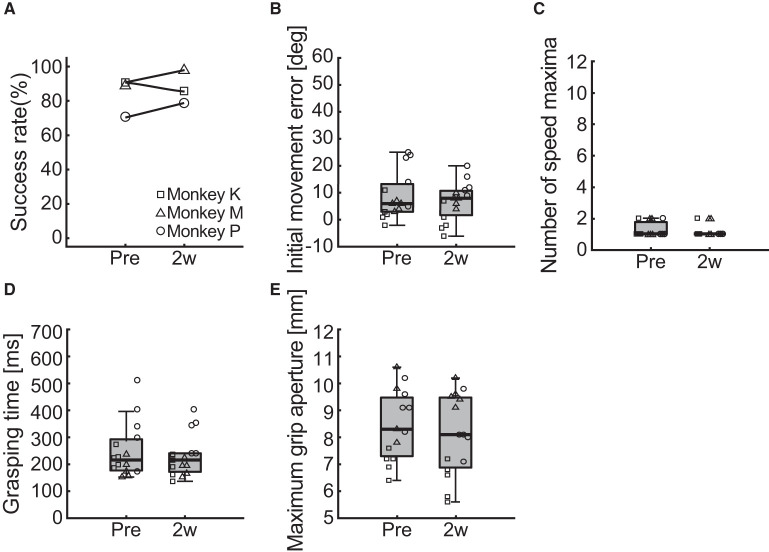
Behavioral results of the ipsilesional forelimb function. **(A)** Changes in the success rate of the pellet-reaching task. **(B–E)** Changes in the indices of reaching and grasping function including initial movement direction error **(B)**, number of speed maxima **(C)**, grasping time **(D)**, and maximum grip aperture **(E)**. Box plots indicate the median (black line in the box), IQR (gray box), and the lowest and highest data within 1.5 IQR of the lower and upper quartile (error bars), respectively.

We observed that the impaired feed-forward and feedback control for the reaching movement did not recover, even 8 weeks after creation of the lesion. To test whether the grasping movement after reaching the target follows the same time course of recovery, we analyzed the grasping kinematics and their changes (Figure 6). Examples of typical grasping movements in one marmoset are shown in Figure 6A (Monkey M). A smooth continuous motion of finger extension (Figure 6Aa), finger flexion (Figure 6Ab) and wrist supination (Figure 6Ac) was observed at “Pre”. However, at “4 weeks,” we noticed a larger grip aperture and clumsiness in the hand-closing movement, although the animal was able to open and close the affected hand (Figure 6Ad, e, f). At “8 weeks,” the grasping movement recovered, and the smooth hand-closing movement was restored (Figure 6Ag, h, i). To characterize these observations, we analyzed the total grasping time from the start to the end of the grasping motion and found a significant interaction between “time point” and “subject” (two-way ART-ANOVA, *F*_(4, 36)_ = 4.06, *p* = 0.0081). However, all animals showed significantly longer grasping times at “4 weeks” (Figure 6B; Monkey K: *p* < 0.0001, Monkey M: *p* < 0.0001, Monkey P: *p* = 0.002) and “8 weeks” (Figure 7B; Monkey K: *p* = 0.0142, Monkey M: *p* = 0.0016, Monkey P: *p* = 0.0003) than at “Pre.” In addition, a similar homogeneous time course of recovery was also observed in the maximum grip aperture (two-way ART-ANOVA, “Time point” × “Subject” interaction: *F*_(4, 36)_ = 1.95, *p* = 0.1236, “Time point” main effect: *F*_(2, 36)_ = 37.1, *p* < 0.0001). Maximum grip aperture was significantly larger at “4 weeks” (Figure 6C; *p* < 0.0001) and “8 weeks” (Figure 6C; *p* = 0.0230) than at “Pre”, and significantly decreased from “4 weeks” to “8 weeks” (Figure 6C; *p* < 0.0001). These findings indicated that the time course of the recovery of the grasping movement differed from that of the reaching movement. Although the animals did not recover their reaching movement, all animals partially recovered their grasping movement 8 weeks after creation of the lesion.

### Control experiment

To test whether the photochemically induced cerebral infarction only affected the reach and grasping functions of the contralesional limb, we performed the same behavioral assessments on the ipsilesional limb. Two measurements were performed before and 2 weeks after creation of the lesion in each monkey and compared. The change in the success rate of the pellet-reaching task is provided in Figure 7A. We found no difference in the success rate between pre- and post-surgery.

Figures 7B–E shows the results of the comparisons of the reaching and grasping function indices of initial movement direction error, number of speed maxima, grasping time, and maximum grip aperture. We found no differences between before and 2 weeks after creation of the lesion for initial movement direction error (Figure 7B; two-way ART-ANOVA, “time point” × “subject” interaction: *F*_(2, 24)_ = 2.03, *p* = 0.1526; “time point” main effect: *F*_(1, 24)_ = 2.61, *p* = 0.1192), number of speed maxima (Figure 7C; two-way ART-ANOVA, “time point” × “subject” interaction: *F*_(2, 24)_ = 0.02, *p* = 0.9793; “time point” main effect: *F*_(1, 24)_ = 0.19, *p* = 0.6642), grasping time (Figure 7D; two-way ART-ANOVA, “time point” × “subject” interaction: *F*_(2, 24)_ = 0.35, *p* = 0.7092; “time point” main effect: *F*_(1, 24)_ = 0.56, *p* = 0.4621), or maximum grip aperture (Figure 7E; two-way ART-ANOVA, “time point” × “subject” interaction: *F*_(2, 24)_ = 2.39, *p* = 0.1132; “time point” main effect: *F*_(1, 24)_ = 1.34, *p* = 0.2588). These results indicated that the lesion only affected the forelimb function of the contralesional, but not the ipsilesional, side.

## Discussion

### Advantages of the present model

In this study, we used a photochemically induced cerebral infarction model. Other primate stroke models, such as occlusion of the middle cerebral (54) and anterior choroidal arteries (26), have variable ischemic lesions because of anatomical variations in the vascular architecture. Variability of lesions inevitably leads to variability in outcome measures, including lack of deficits, and highly reproducible lesions enable the prediction of motor deficits and lower variability of outcome measures (6). All the animals in the current study showed a lesion in motor-related cortical areas and exhibited more or less homogeneous motor impairment of reaching and grasping movements. Therefore, our animal model was more reliable and reproducible in terms of the motor disability. Moreover, most recovery of function was observed within 4 weeks of the lesion creation. For the time course of recovery, our model was comparable to human stroke patients, in whom the most dramatic recovery in motor function occurs during the first 30 days (55).

Although kinematic analysis to predict functional recovery after stroke has been used in human research (56), photothrombosis models in NHPs have never applied kinematic analysis to examine upper limb motor function (36–38). We found that the kinematic aspects of motor impairment were similar across humans and marmosets, as evaluated by kinematic indices commonly used for evaluating human stroke recovery (i.e., initial movement direction errors and the number of speed maxima). According to previous studies (57, 58), reaching movements can be broadly separated into two components: initiating movements (feed-forward control) and corrective movements (feedback control). The former is attributed to initial movement direction errors and the latter to the number of speed maxima. Therefore, we suggest that photochemically induced cerebral infarction is advantageous for reproducing the upper limb motor function impairment seen in human stroke survivors in an NHP model.

### Initial movement direction errors

We observed an increase in initial movement direction errors after creation of the lesion. Previous human (45, 53, 59, 60) and macaque monkey (61) studies have shown comparable impairments in the initial phase of the reaching movement after stroke. Our results indicated that a lesion to the sensorimotor cortex causes impairment of the feed-forward control mechanism of upper limb movement.

We suggest that two potential mechanisms underlie cortically generated feed-forward malfunction. A lesion in the M1 may disrupt the cortical pathway involved in sensorimotor transformation, which is essential for reaching planning (62). Previous human and animal studies have shown that a lesion or inactivation of the M1 disrupts not only motor execution but also sensorimotor planning; moreover, these disruptions are dissociable (62–65). This suggests that the M1 integrates somatosensory information about the limb and visual information about the target location to plan movement trajectories (63).

Another explanation is the dysfunction of the premotor cortex (PM). Our model showed a lesion in the PMd, in addition to the M1. In the PMd of marmosets, areas 6DC and 6DR correspond to areas F2 and F7 in macaques, respectively (52, 66, 67). Similar to the PM of macaques, area 6DC in the marmoset has strong connections to the M1 and is involved in the limb movements (52). Area 6DR is part of the parietofrontal network (52), which plays a role in visually guided reaching and grasping (21, 68). Previous marmoset studies have shown that impairment of the parietofrontal network without damage to M1 disrupts the feed-forward aspect of visually guided reaching and grasping (13, 19).

### Number of speed maxima

We demonstrated that the speed maxima were also affected, which is in line with previous reports of human stroke patients (45, 53, 69–71). The number of speed maxima is considered an indirect measure of the efficiency of continuous corrective feedback control action to reach the target (60). Therefore, the increase in the number of speed maxima in the marmoset stroke model may be a clinically relevant outcome measure. The M1 has been proposed as a feedback controller (72, 73). The motor cortex receives somatosensory information from areas 3a, 3b, and 1/2 (51), and ongoing sensory input is used to refine and update descending motor commands (74, 75). In addition, a previous study of marmosets demonstrated strong motor–somatosensory cortical interactions during reaching (76) and suggests that damage to the M1 disrupts the updating of descending motor commands from sensory inputs. Although evidence has shown that sensory input is critical for motor execution, studies focusing on sensorimotor integration following stroke are limited (75). Our model raised interesting questions about the role of sensorimotor integration in motor recovery following stroke.

### Difference between the recovery of reaching and grasping functions

Our result showed a more homogeneous and faster recovery of grasping than reaching function from 4 to 8 weeks after creation of the lesion in all animals. This result is consistent with previous reports on the recovery process of human stroke patients. Numerous measurements have been used to evaluate the recovery of motor function of the upper extremities in stroke survivors, and it is well established that the recovery process varies depending on the measurement (77–79). Among these, grip strength, a simple measure of power grip function, shows the fastest recovery of all the measures of grasping ability and can occur as early as 3 weeks (77). In contrast, the smoothness of trajectory for target-reaching recovers in 5 weeks (80); moreover, the index for accuracy at the endpoint of reaching, such as initial direction error, takes considerably longer (81).

Different recovery speeds for grasping and reaching in both species could be influenced by what extent the cortical control is required to properly execute each movement. Power grip requires the highly synergistic activity of multiple hand muscles (82). We previously reported that hand muscle synergy could be formed by the spinal interneurons (83). In line with this finding, it is known that activation of the sensorimotor cortex is less dominant during more synergistic power grip and more dominant during precision grip that requires individual finger control, in both human and NHPs (84–86). Therefore, power grip could be generated primarily by the contribution of the non-cortical area in the CNS. On the other hand, target reaching is highly dependent on cortical control, and that involves a widely distributed parietofrontal network (21, 68). In human stroke patients, for example, lesion in the parietofrontal cortex disrupts the target-reaching performance significantly (87, 88). Similarly, the experimental lesion on the comparable cortical area affects the target- reaching performance (17–19).

If the power grip could be generated primarily by the non-cortical area, then, it is reasonable to expect a faster recovery after stroke in the cortex. Because the function expected for the lesioned cortical area may be limited, it could be taken over by other areas in the CNS relatively easily. In contrast, because the lesioned cortical area had a significant contribution to the target reaching, it should take a longer period to be taken over, and thus, its slower recovery is also expected.

### Limitations of the present model

One limitation of our model is the structural differences in the CNS between humans and marmosets. The CNS of marmoset is characterized by a lissencephalic brain (89, 90) and a lack of direct corticomotoneuronal projections to the motoneuron pools of distal hand muscles (91, 92) that are crucial for controlling independent finger movements (93, 94). Consequently, marmosets exhibit lower manual dexterity than humans (11, 76, 95). The corticospinal tract is more developed in humans than in marmosets; moreover, humans have greater cortical functional specialization (96), which may affect the time course of recovery of grasping movements. However, our marmoset model provides an advantage over rodents when assessing visually guided reaching movement that is impaired in human stroke patients.

A further technical consideration is related to the varied recovery time courses among animals for the success rate of the pellet-reaching task 8 weeks after creation of the lesion and initial movement direction errors. Specifically, Monkey M exhibited faster recovery for both measurements, which may be related to the extent of the lesion (the lesioned area of Monkey M was smaller than that of other animals; Table 1). As discussed earlier, reaching movements may be more sensitive to cortical lesions in marmosets. Although we controlled the infarction area using irradiation light, the difference in the extent of the lesion may have resulted in a difference in the time course of behavioral recovery.

## Data availability statement

The raw data supporting the conclusions of this article will be made available by the authors, without undue reservation.

## Ethics statement

The animal study was reviewed and approved by Experimental Animal Committee of the National Institute of Neuroscience.

## Author contributions

YS, TU, and KS designed the study. YS, MKu, MKo, and TU performed the study. AK and YS analyzed the data. AK and KS wrote the draft of the manuscript. All authors approved the final version of the manuscript.
